# Transmembrane Protein Alignment and Fold Recognition Based on Predicted Topology

**DOI:** 10.1371/journal.pone.0069744

**Published:** 2013-07-19

**Authors:** Han Wang, Zhiquan He, Chao Zhang, Li Zhang, Dong Xu

**Affiliations:** 1 School of Computer Science and Information Technology, Northeast Normal University, Changchun, People’s Republic of China; 2 Department of Computer Science, Christopher S. Bond Life Sciences Center, University of Missouri, Columbia, Missouri, United States of America; 3 School of Computer Science and Engineering, Changchun University of Technology, Changchun, People’s Republic of China; CSIR-Institute of Microbial Technology, India

## Abstract

**Background:**

Although Transmembrane Proteins (TMPs) are highly important in various biological processes and pharmaceutical developments, general prediction of TMP structures is still far from satisfactory. Because TMPs have significantly different physicochemical properties from soluble proteins, current protein structure prediction tools for soluble proteins may not work well for TMPs. With the increasing number of experimental TMP structures available, template-based methods have the potential to become broadly applicable for TMP structure prediction. However, the current fold recognition methods for TMPs are not as well developed as they are for soluble proteins.

**Methodology:**

We developed a novel TMP Fold Recognition method, TMFR, to recognize TMP folds based on sequence-to-structure pairwise alignment. The method utilizes topology-based features in alignment together with sequence profile and solvent accessibility. It also incorporates a gap penalty that depends on predicted topology structure segments. Given the difference between α-helical transmembrane protein (αTMP) and β-strands transmembrane protein (βTMP), parameters of scoring functions are trained respectively for these two protein categories using 58 αTMPs and 17 βTMPs in a non-redundant training dataset.

**Results:**

We compared our method with HHalign, a leading alignment tool using a non-redundant testing dataset including 72 αTMPs and 30 βTMPs. Our method achieved 10% and 9% better accuracies than HHalign in αTMPs and βTMPs, respectively. The raw score generated by TMFR is negatively correlated with the structure similarity between the target and the template, which indicates its effectiveness for fold recognition. The result demonstrates TMFR provides an effective TMP-specific fold recognition and alignment method.

## Introduction

Transmembrane proteins (TMPs) play crucial roles in cells serving primarily as transporters and receptors. TMPs are related to many serious diseases [1], and they are the biological targets for most drugs currently on market [2]. Although studying TMP structures is imperative for understanding the central physiological processes, and has immediate medical relevance [3], high-resolution structures of TMP remain scarce because they are hard to be solved experimentally. In fact, TMPs represent only less than 2% of total structures in the Protein Data Bank (PDB) [4], even though the number of TMPs has been continuously increasing in recent years. Meanwhile, with a rapidly growing amount of protein sequences generated by next-generation sequencing, the ability to effectively predict TMP structure is in high demand.

Although substantial efforts have been devoted to predicting the protein structure from amino acid sequence for decades, major advances have been made mostly for soluble proteins with little success in TMP structure prediction [5]. In early studies, *de novo* (or *ab initio*) methods [6]–[9] were explored without resorting to homologous proteins of known structures. However, such methods are mainly effective only on small soluble proteins [10] not on TMPs, which are often large. As more and more TMP structures became available, homology-modeling methods were utilized for prediction. For example, Arnold et al. [11] succeeded in modeling Human Transmembrane Protease 3 using remote homology templates. Kelm et al. applied MEDELLER [5] to separately model transmembrane cores and loops. Because G-protein-coupled receptors (GPCRs) are a major target for the pharmaceutical industry, continuous attention is given to their structure modeling yielding several successful solutions [12]–[17]. Notably, a few methods using residue coevolution analysis became available for large TMP structures recently [18], [19]. However, only a small fraction of TMPs have a significant sequence similarity to those solved structures, confirming that homology-modeling methods have significant limitations for general TMP structure prediction. Hence, fold recognition becomes a highly promising approach because it can utilize templates without significant sequence similarities to the target.

Fold recognition has been widely applied to structure prediction for remote homology soluble proteins [20]–[24], but these methods often perform poorly on TMPs because the significant biochemical and biophysical differences between the two types of proteins. Few methods have been customized for TMPs. However, TMP structure prediction has been estimated to obtain accuracy as high as that of soluble proteins if the alignment for TMP achieves the accuracy as its soluble protein counterpart [25]. Some alignment methods for TMP have been developed recently [26], but they generally focus on the cases with significant sequence similarity between the target and the template. New methods using more general alignments are needed. With the increasing number of TMP structures, the features used in fold recognition such as sequence profile and solvent accessibility become more and more reliable to describe the properties of TMPs. Notably, the special spatial conformation of TMPs, which shows much more uniform secondary structures than typical soluble proteins, has underlying advantages to improve the alignment.

TMPs usually span the biological membrane by either all transmembrane alpha-helices (TMH) in αTMP, or all transmembrane beta-strands (TMB) in βTMP. The remaining parts of TMPs are non-TM segments, including inside segment (located in the cytoplasmic side) and outside segment (located in the extracellular side). In most cases, the inside segment and outside segment appear alternatively on a protein sequence, resulting in TM segments having specific orientations. This significant topological feature may potentially improve the TMP fold recognition and has been introduced previously to a few TMP structure studies [27], or even 3D structure modeling of for βTMPs [28], [29].

For a given TMP, topology structure can be predicted by topology predictors from amino acid sequence alone. It is observed that TM segments are highly hydrophobic and regular in sequence length, TMHs are normally between 17 and 25 residues [30], while TMBs have 11 residues on average in trimeric porins and 13–14 residues in monomeric beta barrels [31]. Hydrophobicity scales were widely adopted in early topology predictions [32]–[34]. Utilization of a “positive-inside” rule [35] improved prediction accuracy. Further success was made after machine learning methods were employed for αTMPs, such as Hidden Markov Model (HMM) based methods [36]–[42], neural networks (NN) based methods [43], [44], and support vector machines (SVM) based methods [45], [46]. Furthermore, MemBrain [47] combined numerous machine learning methods together to improve prediction accuracy. However, the prediction accuracy of these methods may be overestimated in whole-genome studies [48], [49]. Comparably, βTMP predictors [50]–[53] mainly rely on amino acid composition and alternating hydrophobicity pattern [54] because fewer sequence patterns can be found for βTMP than for αTMPs; therefore, βTMP predictors are often less accurate than αTMP predictors.

In this study, we developed a TMP Fold Recognition method, TMFR, based on a sequence-to-structure pairwise alignment method. Given that TMPs have distinct topology structures, we first combine the topology-based features, segment type and segment orientation with sequence profile and solvent accessibility to build profiles for each sequence position. Then we design a scoring function to utilize those TMP-specific features where fitness scoring is used to measure the compatibility of two position profiles, and a segment-dependent penalty model is used to further minimize incorrect alignments. In addition, high-accuracy αTMP topology prediction generated by our previous work [55] is used to further improve the alignment accuracy. Tested using a non-redundant TMP dataset, TMFR can accurately align the target sequence to the template structure and generate reliable alignment raw scores to evaluate the structural similarity between target and template. Overall, our method achieved higher accuracy both in alignment and fold recognition than existing leading methods HHalign and HHsearch on the same testing dataset, respectively.

## Materials and Methods

### Datasets

The Protein Data Bank of Transmembrane Proteins (PDBTM) [56] is the most comprehensive TMP database currently available. It uses an automated algorithm (TMDET) [57] to identify TMPs in PDB and calculate their topology structures. Compared to peer databases [58], [59], PDBTM is convenient for large-scale testing, and updated weekly by synchronizing with PDB. Hence, we selected PDBTM as the data source in our study. There were 4447 TMP sequences derived from 1626 TMP entries including 1383 αTMPs and 232 βTMPs at the time of study. We removed the entries if their lengths were less than 50 amino acids or more than 30% of all heavy atoms did not have atomic coordinates. Bitopic TM entries were also excluded. Finally, we selected non-redundant TMPs, in which mutual sequence identity between any two sequences in the datasets were less than 30%. These TMPs were divided randomly into the training dataset and testing dataset. The training dataset contains of 58 polytopic αTMP sequences and 17 βTMP sequences, while 70 and 30, respectively are in the testing dataset (see Table S1, S2).

### Profile Generation

The features extracted from each position on a target amino acid sequence were used to construct a position-dependent profile for alignment. The selected features describe various properties of proteins, and they are expected to have minimum dependency on each other. Hence, we selected a small set of features for TMPs, including features of segment type, segment orientation, sequence profile, and solvent accessibility. Sequence profile and solvent accessibility are widely used in alignment methods, while segment type and orientation are topology-based features, which utilize the TMP’s special conformation. All of these features will be further introduced below.

### Topology-based Features

Topology structures of TMP are often divided into three segment types according to their locations relative to biological membrane, including TM segment, inside segment (inside the area surrounded by biomembranes) and outside segment (outside the area surrounded by biomembranes). Therefore, aligning the target and template using topology segment types can achieve more accuracy than only using secondary structures for TMPs. Meanwhile, the orientation of TM segment, namely from which side it crosses the membrane, can further identify whether two TM segments match.

**Figure 1:**
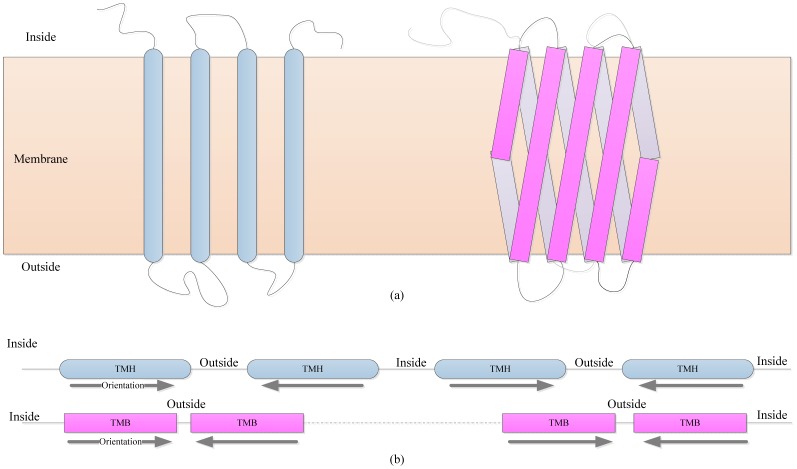
Topology structures of αTMPs and βTMPs. (a) Sketches of native TMPs located in biological membrane, where the left one represents an αTMP, and right one is a βTMP. (b) Linear topology of the two TMPs. TM segments are labeled as TMH or TMB respectively according to their TMP types. Orientations of TM segments are described using arrows.

Topology structure is described as a sequence with the same length of amino acid sequence, where the positions on TM segments are denoted to ‘H’ (TMH), or ‘B’ (TMB), while the ones on non-TM segments are ‘O’ (Outside segment) or ‘I’ (Inside segment), and others are ‘U’ (Unknown). An αTMP is located in biological membrane as shown in Fig. 1(a) left, and a βTMP is shown in right. Their topological structures are presented in Fig. 1(b), where TM segments, non-TM segments and orientations of TM segments are labeled. To facilitate the calculation, the segment orientations are denoted 0, 1, and −1, respectively for non-TM segments, TM segments that span membrane from outside to inside, and other TM segments pointing toward the opposite direction.

The alignment accuracy strongly depends on the reliability of predicted topology structures. We used predicted topological structures to derive features for both the target and the template, since the features used between the target and the template are more likely to be consistent than using those derived from predicted topological structure of the target but the known topological structure of the template. Furthermore, we used our consensus topology predictor CNTOP [55] to generate highly accurate topology structures for αTMP, in which contact of TMH residues is utilized to improve the topology prediction accuracy based on four top-leading individual predictors. By using the same training and testing sets as the one used in the current study, CNTOP achieved 87% prediction accuracy and located TMHs more accurately than any individual predictor. Although the topology prediction for βTMP is not as accurate as αTMP because they often have shorter TM segments and less sequence pattern, these barrel TMPs have more regular and simple global topology structures than their TMH counterparts; in particular, βTMPs in the same fold have mostly the same number of TM segments and similar sequence lengths. Therefore, the current topology prediction accuracy of βTMP is still very useful to generate a reliable alignment. TMBETAPRED-RBF [53] was used as βTMP topology predictor for its higher prediction accuracy.

### Sequence Profile

To get sequence profile for a given protein sequence, we used the Position Specific Scoring Matrix (PSSM) derived from the search of PSI-BLAST (Position Specific Iterative BLAST) [60] against NCBI’s non-redundant (NR) database. A PSSM profile 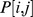 is a 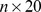 log-odds matrix, where the
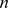
represents the sequence length. Each element in 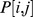 indicates the frequency of the residue type 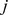 appearing at position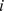.

### Solvent/Lipid Accessibility

Accessible surface area (ASA) describes a residue’s exposure to the environment, and it has been applied to structural studies of soluble proteins [20], [61]–[63]. A number of ASA predictors have been developed [24], [64]. In contrast, TMPs interact with not only a hydrophilic solvent environment (non-TM segments), but also a hydrophobic lipid environment (TM segments). The average ASA of 20 amino acids in TMPs are significantly different from that of soluble proteins, even in non-TM segments [65]; hence, ASA predictors of soluble proteins are not applicable to TMPs. However, some studies on predicting ASA specifically for TMPs [65]–[67] have been developed, which showed significantly improved accuracy of ASA prediction in TM segments. We used one of these methods MPRAP [67] to predict ASA for both targets and templates, which separates different segments of TMP and predicts the entire TMP sequence without using its topology structures as input. To reduce the impact of prediction errors in the alignment, both the target and template used predicted ASA to construct profiles.

### Scoring Function

We employed a scoring function consisting of fitness score with gap penalty, where the fitness score was used to measure the compatibility of the profiles between the target and the template, while the gap penalty minimized gap insertions in alignment. Scoring function applied to our method was tailored for TMPs based on their special topology structures as shown in the following.

#### (1) Fitness scoring

Fitness scoring used in our method compares the compatibility of profiles constructed by the four integrated features. The fitness scoring between position
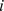
on the target and position 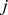 on the template is given as follows:
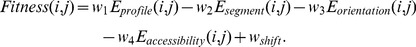
(1)


The first term of Eqn. (1) describes the compatibility of sequence profiles between the target position 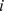 and the template position 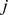, which is calculated as follows:
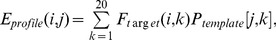
(2)where 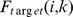 is the sequence-derived frequency of residue 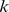 at position 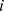 on the target sequence, and 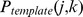 is the PSSM value of residue 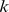 at position 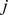 on the template sequence.

The second term presents the match score of segment type between two positions, i.e.
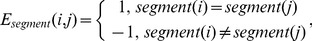
(3)where 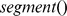 represents the segment type of the residue at the corresponding position. Both the target and template use segment type derived from predicted topology structures.

The third term is used to further distinguish the TM segments by segment orientation, which is given as,
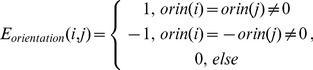
(4)where 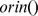 is the segment orientation of the residue at the corresponding position. The TM segments that have the same orientation obtain 1, while the opposite orientation results in −1. The score between TM segment and non-TM segment is assigned to 0, because such a comparison is not taken into consideration.

Similarity of accessibility between positions 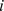 and 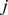 is measured as,
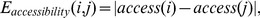
(5)where 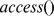 is the real value of predicted accessibility of the residue at the corresponding position. 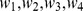 are the weights of four features, and 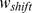 is a to-be-determinate constant shift [68], which was trained with other parameters.

#### (2) Gap penalty

Gap penalty is used to evaluate the cost of an insertion (or deletion) in the alignment. We employed a segment-dependent gap penalty model, which is composed of open gap penalties 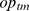,
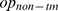
_,_ and extended gap penalties 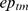,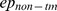 for TM segments and non-TM segments, respectively. Differing from an early study [69] which simply forbade the gaps opening inside alpha-helices and beta-strands, we still allow gaps to open in TM segments because topological structure prediction may have prediction errors. On the other hand, open gap penalty for TM segments is significantly larger than that of non-TM segment.

#### (3) Alignment score adjustment

The raw score generated by alignment, which is the score of optimized dynamic programming path, is used to rank the templates for a given target from which best matching folds are selected, the lower raw score is, and the better alignment was made. However, raw score is sensitive to the sequence lengths of the target and the template. Hence, we adjust the raw scores according to the sequence length difference between target and template as follows:
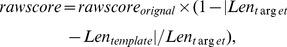
(6)where 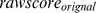 is the original alignment score, and 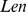 is the sequence length of target or template. This score favors the alignment between a target and a template of similar lengths.

### Dynamic Programming

We used a local-global dynamic programming (DP) algorithm [70] to optimize the alignment path, together with the OMP-specific scoring function introduced above. The segments with the same type are favored in the alignment, while different segment types are hard to match unless they are highly compatible with the sequence profiles.

### Training of Parameters

All parameters,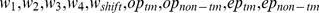 used in the scoring function were trained using the method in [69] on our training dataset for αTMP and βTMP separately. All the parameters were randomly assigned the initial values, and then optimized by a grid search. Here, the TM-Score [71] was used to guide the searching. The higher TM-Score derived from the alignment is considered achieving a higher accuracy. The iterations exit when the average TM-Score stopped increasing. The parameters trained for αTMP are (1.6, 8.4, 6.7, 3.2, 4, 12.1, 1.6, 8.6, 1.1), and those of βTMP are (1.5, 9.2, 4.3, 3.6, 5, 9.2, 11.8, 1.6, 8.3, 1.1).

### Benchmarks

The alignment accuracy can be evaluated by two approaches: (1) calculating the percentage of correctly aligned positions [72]; (2) scoring the structural similarity between the aligned pairs [73]. A ‘ground truth’ benchmark is required for both approaches. For the first one, reliable native 3D structure alignment is used to identify the correct aligned positions and the alignment accuracy (ACC) is recorded. While there is no unique solution that solves the problem of finding the optimal structure alignment [74], we chose TM-align [75] for such a golden standard given its good performance. For the second approach, GDT_TS [76], [77] and TM-score [71] are commonly used for alignment purposes, and we used both of them to fully assess the alignment accuracy of TMFR. Notably, TM-score is designed to be independent of protein lengths, and the structures with a score higher than 0.5 assume the same fold, while the proteins are assumed unrelated when the score is below 0.20 [78]. Since there is no comprehensive fold classification database that involves all the TMPs, we used TM-scores to determine whether two TMPs are the same fold using a threshold of 0.5.

## Results

### Performance of Alignment

Since there is no existing alignment method specifically for TMP to make comparison, we used HHalign [79], which is a leading alignment method for general proteins, to compare the performance of alignment. HHalign uses profile hidden Markov model (HMM) to make pairwise HMM-HMM (profile-HMM) alignments, where confidence values and a full seven-state secondary structure prediction are employed to improve the alignment quality.

To arrange the comparison, the profile-HMMs of all TMPs in the testing dataset were generated with default parameters and then applied to an all-vs-all pairwise alignment using HHalign. Self-alignment of the same protein, and alignments between αTMPs and βTMPs were removed. In total, 5700 pairs (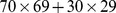) were used in the final comparison. Correspondingly, the same pairwise alignment was made using TMFR alignment on the same dataset.

**Figure 2:**
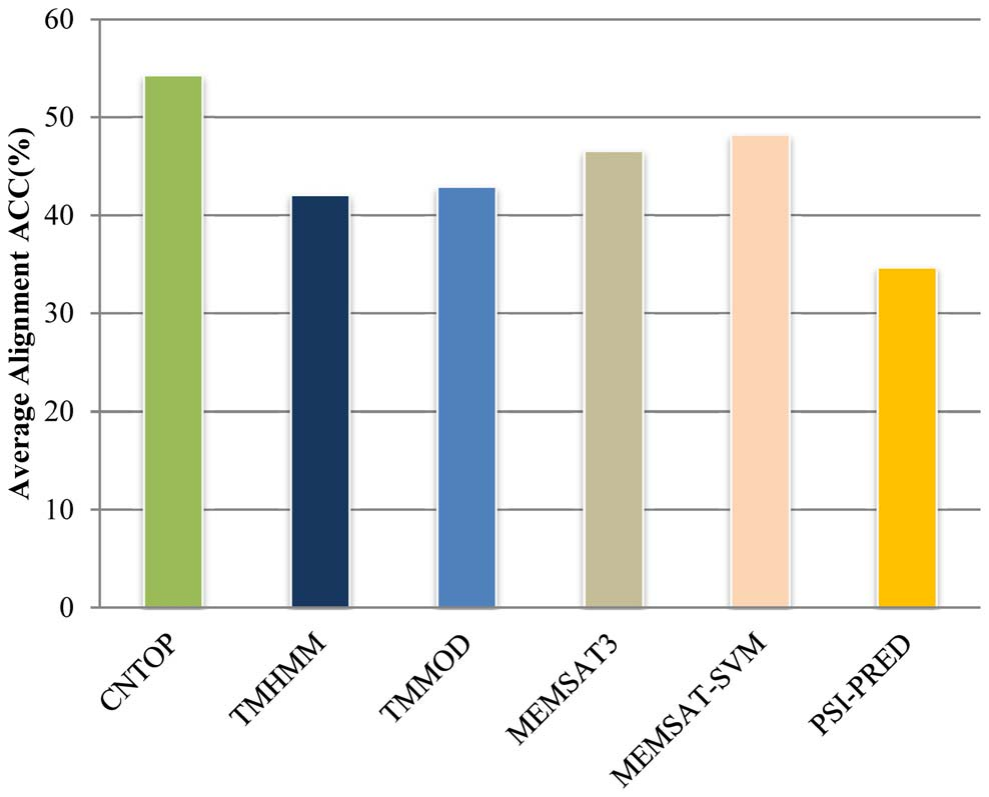
Alignment accuracy by using topology structure or secondary structure. The topology structure improves the alignment accuracy of TMFR^α^ (TMFR for αTMPs) comparing with secondary structure, where CNTOP, TMHMM, MEMSAT3 and MEMSAT-SVM were used to general topology structure features, and PSIPRED was for secondary structure feature. TMFR^α^ derived the best alignment accuracy by using CNTOP, which produced more accurate topology structure prediction than other predictors.

Average alignment accuracies obtained from TMFR and HHalign are shown in Table 1, where αTMPs and βTMPs are separately compared. TMFR achieved better alignment accuracy for both αTMP and βTMP, especially in TM segments. TMFR achieved above 10% improvement on overall ACC over HHalign for αTMP, and 9% for βTMP. Similar improvement was shown using TM-score and GDT_TS, where overall accuracies improved by almost 10% for both categories of TMPs. Notably, TMFR aligned TM segments much better than non-TM segments, and the difference is more significant for αTMPs, while HHalign has a similar pattern, but to a much lesser degree. The better performance in TM segments for both methods may be due to topology-based features and stronger sequence profiles in the regions. We also compared the performance of TMFR between using topology structure and using secondary structure as shown in Fig. 2. Five αTMP topology predictors [37], [38], [44], [46], [55] and one secondary structure predictor [80] were applied to generate corresponding features. The results obviously prove that topology structure was more effective as features than secondary structure for the alignment, and the alignment accuracy increased with the rising topology prediction accuracy. HHalign uses secondary structures as a feature, while TMFR uses richer features of segment type and orientation to represent the conformation of TMPs. This may be the main reason why TMFR achieves significantly better alignment accuracy than HHalign.

**Table 1:** Average alignment accuracy of TMFR compared to HHalign.

Methods	ACC (%)			TM-score			GDT_TS		
	TM	Non-TM	Overall	TM	Non-TM	Overall	TM	Non-TM	Overall
TMFR^α^	55.3	43.1	54.3	0.417	0.282	0.376	0.382	0.216	0.325
TMFR^β^	52.3	46.2	50.2	0.411	0.312	0.363	0.393	0.204	0.317
HHalign^α^	45.9	43.6	44.1	0.267	0.313	0.281	0.223	0.247	0.238
HHalign^β^	43.3	38.6	41.2	0.253	0.275	0.264	0.208	0.212	0.209

ACC is the alignment accuracy according to TM-align. The comparison is made separately in TM segments, non-TM segments and overall proteins.

### Raw Score and Structure Similarity

**Figure 3:**
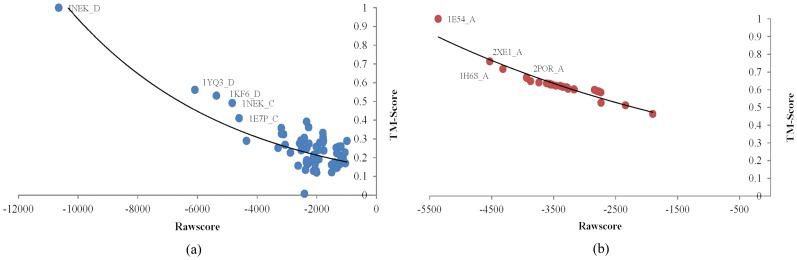
Examples showing the correlation of raw score and structure similarity between target and template. The example of αTMP 1NEK_D is shown in (a), and that of βTMP in (b). Each point on the diagram represents an aligned template. The horizontal axis represents aligned raw score, and the vertical axis shows the corresponding TM-Score. The curve on the diagram is the trend line of data points. The Pearson Correlation Coefficient of 1NEK_D is −0.8120, and that of 1E54_A is −0.8350. Structure similarity is represented using TM-Score. The raw scores generated by TMFR were observed negatively correlating to structure similarities of templates aligned to corresponding target. The templates that have the most similar structures with target are labeled using the PDB classification.

**Figure 4:**
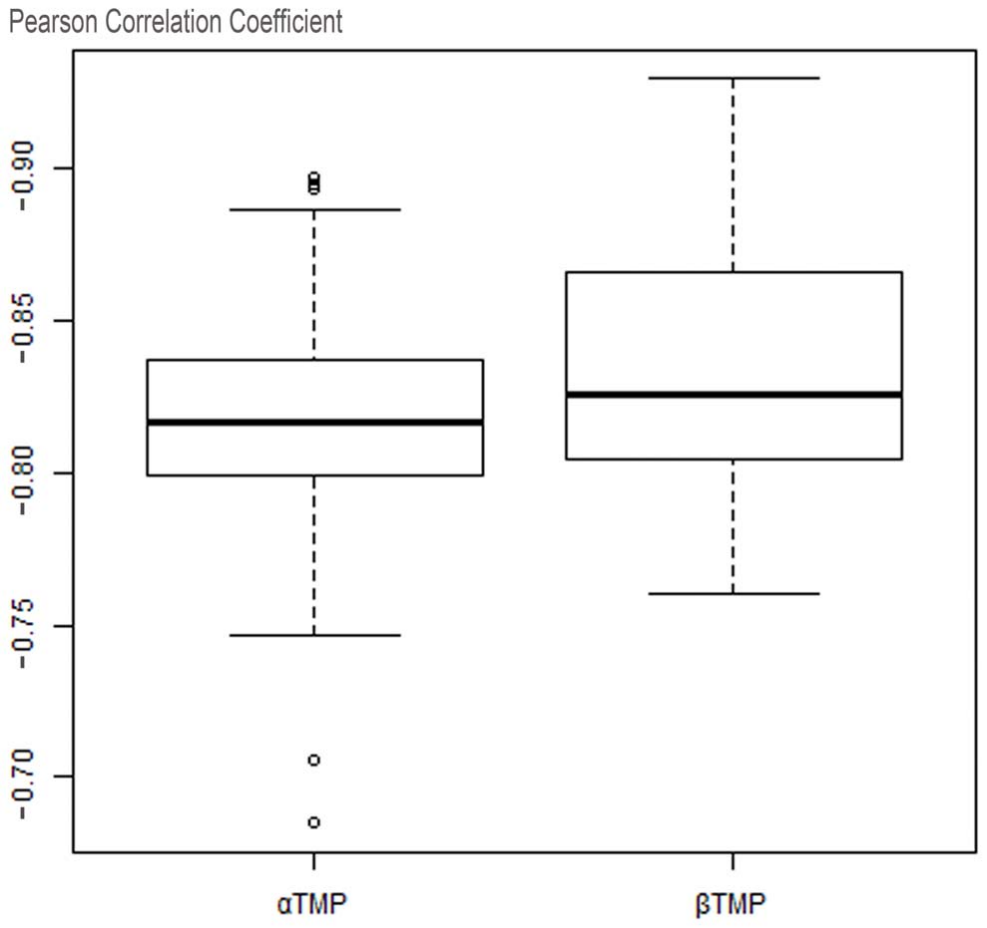
Correlation of raw score and structure similarity in complete testing dataset. The Pearson’s correlation coefficients of αTMP and βTMP samples are separately counted in the boxplot above.

As introduced, TMFR recognizes TMP folds using the ranking of alignment raw scores; hence, how raw score correlates with the structure similarity is the basis of fold recognition. Figure 3 shows two examples where the raw score negatively correlates the structure similarity between the template and the target. Figure 3(a) presents an example of αTMP Succinate Dehydrogenase (PDB_ID: 1NEK:D) [81], and Fig. 3(b ) shows βTMP Omp32 (PDB_ID: 1E54:A) [82]. Both target proteins are selected randomly from the testing dataset and represent typical cases of tested targets, and the distributions of Pearson’ correlation coefficients of αTMP and βTMP are shown together in Fig. 4, which indicts how the raw score produced by TMFR is relative to structure similarity.

**Figure 5:**
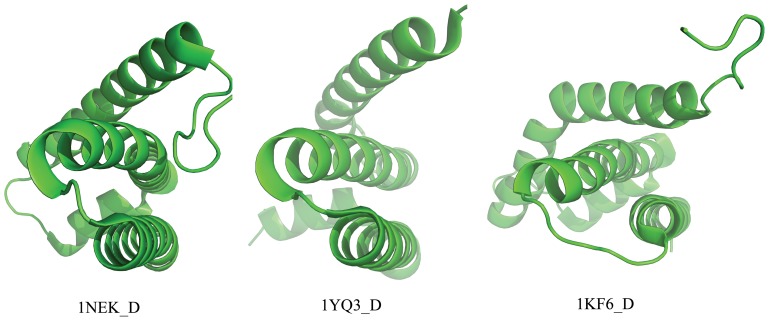
Topological arrangements of top-ranked templates for target 1NEK_D. 1YQ3_D and 1KF6_D are the top-2 templates ranked by raw score.

As expected, the targets yielded the best raw scores (smallest) when they aligned to themselves as shown by the data points in the graph’s left-top area. In the case of 1NEK_D, templates with structural similarity less than 0.4 of TM-Score cluster in the graph’s right-bottom area, while a few templates fall in the middle area, e.g., mitochondrial respiratory Complex II (1YQ3_D) [83] and *Escherichia coli* quinol-fumarate reductase (1KF6_D) [84]. These protein domains having high raw scores also have the similar topological arrangement as shown in Fig. 5. The trend line clearly indicates that the distribution of templates reflects the tendency that raw scores are negatively correlated with their structural similarities to the target protein. Although the ranking of raw scores does not always follow the structure similarities, especially for the templates with low TM-Scores, the templates in the same fold with target (TM-Scores>0.5) have more significant correlation, which is more relevant for fold recognition.

In contrast, the trend line of βTMP target 1E54_A demonstrates more correlation than 1NEK_D between raw scores of templates and their structure similarities to the target as shown in Fig. 3(b). The three templates, namely, OmpC (PDB_ID:2XE1:A) [85], engineered porins (PDB_ID:1H6S:A) [86] and porin (PDB_ID:2OPR:A), have the most similar structures with target, and they all have 16 TMBs same as 1E54_A. As βTMPs are often homologous to each other [87], βTMPs having the same number of TMBs are more likely to result in similar spatial structures. This may be why βTMP templates derive much higher TM-Scores with the target than 0.4, while most αTMP templates have less than 0.4 TM-Scores to their target. It is noted that good correlation shown in Fig. 3(b) does not cover all βTMPs even when having the same number of TMBs between the target and templates.

### Performance of Fold Recognition

Given the absence of available method for TMP fold recognition, HHsearch [79], a leading fold recognition program based on the profile-HMM pairwise alignment method, HHalign, was used to compare with TMFR. On the same testing dataset, templates were ranked using the raw scores generated previously in the above subsection in αTMP and βTMP separately. The performance of both methods is shown in Table 2. TMFR achieved better accuracy of fold recognition in all aspects compared to HHsearch. TMFR improved the top-1 βTMP fold recognition nearly 11% more than HHsearch in average accuracy, and improved over 7% in top-1 αTMP fold recognition. When both methods recognized the top-1 template correctly at the fold level (TM-Score>0.5), the top-1 templates ranked by TMFR usually have closer structures to the target than HHsearch. Meanwhile, TMFR performed even better in recognition of top-3 templates, where the average accuracy gap between the two methods was ∼9% for both αTMP and βTMP, as indicated by the average TM-Score.

**Table 2:** Comparison of fold recognition performances between OMPs and HHsearch.

Methods	Top 1		Top 3	
	ACC. (%)	TM-Score	ACC. (%)	TM-Score
TMFR^α^	56.3	0.581	66.7	0.523
TMFR^β^	93.1	0.738	93.6	0.684
HHsearch^α^	49.2	0.553	57.2	0.467
HHsearch^β^	82.8	0.692	84.5	0.603

Average accuracy (ACC) is the percentage of correctly recognized templates for all tested targets, where, a template has been correctly recognized when its structure similarity and raw score have both ranked in the top-1 (or top-3), and “TM-Score” is for top-1 template or the average of top-3 templates.

## Discussion and Conclusion

In this study, we developed a TMP fold recognition method, TMFR, which employs topology-based features to improve the pairwise alignment using the distinct physicochemical properties of TMPs compared to soluble proteins. We further introduced the TM segment orientation to distinguish the TMPs with similar topology structures. Compared with a leading general protein fold recognition method, HHsearch, TMFR achieved significant improvements both in pairwise alignment and fold recognition. Our study shows that TMP-specific features can benefit the sequence-to-structure alignment significantly, which provides some insight for future structure prediction and function annotation for TMPs.

Our current study has some limitations and future work will address them. The performance of TMFR heavily relies on topology structure prediction whose advance will help TMP fold recognition and alignment. In addition, topology structure does not include the secondary structures within non-TM segments. Integrating secondary structures of non-TM segments with topology structures of TM segments may improve our method in the future. We will also develop a web server for the broad research community.

## Supporting Information

Table S1 Training dataset.
(DOCX)

Table S2 Testing dataset.
(DOCX)
