# Supplementary material for: Transmembrane Protein Alignment and Fold Recognition Based on Predicted Topology
Source: PLoS One. 2013 Jul 19;8(7):e69744. doi: 10.1371/journal.pone.0069744 (PMC3716705; doi:10.1371/journal.pone.0069744)
Supplement: Table S1 — Training dataset. (DOCX) [file pone.0069744.s001.docx]

**Table S1. Training dataset**.

| αTMP | 2WJM_M 2JAF_A 3CN6_A 1KPL_A 3EFF_K 1M56_A 3M72_A 2WIE_A 1M56_B 2IC8_A 2NS1_A 2BG9_B 3EHZ_A 1OED_E 1Y5I_C 2FBW_C 1XME_A 3LNM_B 3KZI_C 2YXQ_A 3BHS_A 2J4Y_A 3A0B_A 2PNO_A 2J8S_A 3BA6_A 3B8C_A 3A0B_B 3D4S_A 2QTS_A 2ZIY_A 3GI9_C 2V8N_A 3FI1_A 2QI9_A 3H90_A 2ONK_C 2K4T_A 3KLY_A 1JB0_L 2F93_B 2DB4_A 1ZOY_D 3HFX_A 2JLN_A 1WP1_A 3I5D_A 3A2R_X 1XL4_A 1OKC_A 1ORQ_C 1FFT_C 2KSE_A 2OAR_A 2KSD_A 2KSF_A 1S5L_Z 1H2S_B |
| --- | --- |
| βTMP | 2J1N_A 2FCP_A 2GE4_A 3M3R_A 1XKH_A 2FGQ_X 2GR7_A 1PNZ_A 1AF6_A 1OH2_Q 2K4T_A 2JQY_A 2WJR_A 3GP6_A 1WP1_A 3A2R_X 1TQQ_A |
