# Supplementary material for: Transmembrane Protein Alignment and Fold Recognition Based on Predicted Topology
Source: PLoS One. 2013 Jul 19;8(7):e69744. doi: 10.1371/journal.pone.0069744 (PMC3716705; doi:10.1371/journal.pone.0069744)
Supplement: Table S2 — Testing dataset. (DOCX) [file pone.0069744.s002.docx]

**Table S2. Testing dataset**.

| αTMP | 1EYS_M 3A7K_A 3CN5_A 1KPK_A 2A9H_A 1FFT_A 3M71_A 1YCE_A 1AR1_B 2NR9_A 2NUU_A 2BG9_A 3EAM_A 1OED_C 1Q16_C 1YQ3_C 3EH3_A 2R9R_B 1S5L_C 1RHZ_A 3B9W_A 1GZM_A 1S5L_A 2Q7M_A 2GIF_A 2ZXE_A 1IWO_A 1MHS_A 1S5L_B 2RH1_A 3HGC_A 2Z73_A 3GI8_C 2K9P_A 1VRY_A 3DIN_E 1PV6_A 1ZCD_A 1L7V_A 2QFI_A 3D31_C 3KCU_A 3CHX_B 2WSC_L 2F95_B 2BL2_A 1YQ3_D 2HYD_A 2QJU_A 2YVX_A 2ZJS_Y 1YEW_A 2GFP_A 2ZD9_A 2VL0_A 1FFT_B 2WCD_A 3CHX_C 3B4R_A 1KQF_C 3DHW_A 2K73_A 1C17_M 2KSR_A 3DWW_A 1ORS_C 1KF6_C 1NEK_C 2KDC_A 1KF6_D 1NEK_D 3DL8_E |
| --- | --- |
| βTMP | 2XE1_A 1H6S_1 1T16_A 1QFF_A 2K0L_A 3M2L_A 2IAH_A 1E54_A 3EMO_C 3CSL_A 1KMO_A 1MPR_A 1A0S_P 3EMN_X 2IWV_A 2WJQ_A 1MM4_A 3KVN_X 2GSK_A 3DWO_X 2O4V_A 3JTY_A 2POR_A 1I78_A 3FID_A 1K24_A 1UUN_A 1P4T_A 2ERV_A 1FW2_A |
